# Within a smoking-cessation program, what impact does genetic information on lung cancer need to have to demonstrate cost-effectiveness?

**DOI:** 10.1186/1478-7547-8-18

**Published:** 2010-09-16

**Authors:** Louisa G Gordon, Nicholas G Hirst, Robert P Young, Paul M Brown

**Affiliations:** 1 Queensland Institute of Medical Research, Genetics and Population Health Division, PO Royal Brisbane Hospital, Herston Q4029, Australia; 2 Department of Medicine, Auckland Hospital, Private Bag 92019, Auckland, New Zealand; 3 School of Population Health, The University of Auckland, Cnr Morrin & Meriton Rds, Glen Innes, Auckland 1142, New Zealand

## Abstract

**Background:**

Many smoking-cessation programs and pharmaceutical aids demonstrate substantial health gains for a relatively low allocation of resources. Genetic information represents a type of individualized or personal feedback regarding the risk of developing lung cancer, and hence the potential benefits from stopping smoking, may motivate the person to remain smoke-free. The purpose of this study was to explore what the impact of a genetic test needs to have within a typical smoking-cessation program aimed at heavy smokers in order to be cost-effective.

**Methods:**

Two strategies were modelled for a hypothetical cohort of heavy smokers aged 50 years; individuals either received or did not receive a genetic test within the course of a usual smoking-cessation intervention comprising nicotine replacement therapy (NRT) and counselling. A Markov model was constructed using evidence from published randomized controlled trials and meta-analyses for estimates on 12-month quit rates and long-term relapse rates. Epidemiological data were used for estimates on lung cancer risk stratified by time since quitting and smoking patterns. Extensive sensitivity analyses were used to explore parameter uncertainty.

**Results:**

The discounted incremental cost per QALY was AU$34,687 (95% CI $12,483, $87,734) over 35 years. At a willingness-to-pay of AU$20,000 per QALY gained, the genetic testing strategy needs to produce a 12-month quit rate of at least 12.4% or a relapse rate 12% lower than NRT and counselling alone for it to be equally cost-effective. The likelihood that adding a genetic test to the usual smoking-cessation intervention is cost-effective was 20.6% however cost-effectiveness ratios were favourable in certain situations (e.g., applied to men only, a 60 year old cohort).

**Conclusions:**

The findings were sensitive to small changes in critical variables such as the 12-month quit rates and relapse rates. As such, the cost-effectiveness of the genetic testing smoking cessation program is uncertain. Further clinical research on smoking-cessation quit and relapse rates following genetic testing is needed to inform its cost-effectiveness.

## Background

Smoking remains a substantial health problem in many countries and is the largest modifiable risk factor for several cancers and a host of chronic diseases. Between 1980 and 2004, smoking prevalence in the Australian population dropped from 40% to 21% [1] partly due to progressive tobacco control policies such as cigarette taxation, smoke-free workplaces and extensive public education campaigns. However, smokers remain a large proportion of the population (21%) as in other European countries (around 30%)[2]. It has been proposed that while system-level public health approaches are effective at reducing aggregate smoking levels, a 'one size fits all' approach may not be effective for all types of smokers[3].

The pivotal paper by Cromwell J *et al*. (1997) demonstrated the cost-effectiveness of smoking-cessation programs delivered by a general practitioner (GP)[4]. Many subsequent smoking-cessation programs have also demonstrated substantial health gains for a relatively low allocation of resources[5]. However, despite being cost-effective, smoking-cessation programs still suffer from low success rates in terms of numbers of quitters at 12-months. As a general guide, the 12-month quit rates are around 6% for brief GP advice, 9% for proactive counselling, 6-12% for nicotine replacement therapies with counselling, and 12-19% for pharmacotherapies with counselling[6]. The extent of relapse following successful smoking-cessation further erodes their effectiveness. This suggests that many smokers may require other measures, such as targeted or personalised information, to encourage cessation and abstinence.

While tobacco smoking is the largest known risk factor for lung cancer occurring in 85-90% of cases, only 10-15% of smokers develop lung cancer[7]. Recent evidence suggests that this may be partly due to differences in genetic susceptibility to lung cancer[7,8]. That is, the smoking-gene interaction means that some smokers are at greater risk of developing lung cancer, with several host characteristics (i.e., *K-ras, GSTM1, CYP2D6, c-MET, NKX2-1, LKB1, BRAF*) implicated in lung cancer onset [9]. Further, other genes are implicated in other chronic diseases linked with smoking, therefore smoking-cessation has wider health benefits and therefore is always beneficial.

The genetic link to lung cancer has implications for the design of smoking-cessation programs. Genetic information represents a type of individualized or personal feedback regarding the risk of developing lung cancer, and hence the potential benefits from stopping smoking, may motivate the person to remain smoke-free. Central to this is the potential to address the issue of optimistic bias, the underestimation of one's own risk of a harmful outcome relative to the average smoker. Recent developments in genetics suggests that some people respond well to genetic information about risk of lung cancer [10,11], are more likely to quit [12] and perhaps less likely to relapse. Combining a genetic test with a smoking-cessation program might enhance the effectiveness and thus represent a cost-effective intervention.

Several companies now offer genetic testing for lung cancer susceptibility however they offer a single nucleotide polymorphism (SNP) test for lung cancer risk result and no other clinical data is used for their risk assessment. Our author (R.Young) heads a clinical research program at Auckland Hospital, New Zealand, offering patients a SNP-based test involving 20 SNPs and assessment of other clinical variables (family history, COPD, smoking patterns) within usual clinical practice for smoking-cessation. Early results show that intentions to quit smoking among 250 participants based on genetic testing for lung cancer risk were around 88% in those at elevated risk of lung cancer. The economic value of the adopting this new technology into practice is yet to be determined.

To date, no smoking-cessation study has examined the cost-effectiveness of offering genetic tests in the context of disease prevention but other studies have investigated genetic testing to guide the choice of pharmacotherapy among individuals attempting to stop smoking [13,14]. Genetic testing imposes costs on individuals, doctors and the health system. Thus, if genetic testing is to be offered in addition to a first-line smoking-cessation program, then it must result in enough new quitters (or reduced numbers of relapsers) in order to justify the costs. The purpose of this study was to explore how much of an impact genetic testing information would need to have in order to be a cost-effective addition to a typical smoking-cessation program. Specifically, we assess the net costs, and health benefits of a smoking-cessation program with a genetic test compared with nicotine replacement smoking-cessation treatment.

## Methods

### Markov model structure

**Figure 1:**
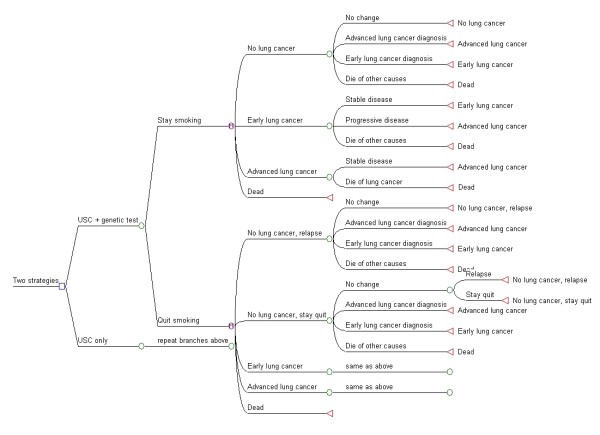
**Illustration of Markov Model**.

A Markov state transition model was constructed in TreeAge Pro 2009 software (TreeAge Software Inc, Williamstown, MA, USA) (Figure 1). The model, known as a Markov single cohort model, is cyclical, with patients moving between specified health states at the end of each cycle, with subsequent cost and quality of life implications. The advantage of this type of model is that it explicitly identifies the sequence and linkage of events under consideration and allows detailed analyses on data parameters. Two decision strategies were modelled; individuals either received or did not receive a genetic test component within the course of a usual smoking-cessation intervention. The model tracked a hypothetical cohort of smokers over 35 years from age 50 who faced different probabilities of quitting smoking, risk of developing lung cancer and transferring between different health states (Table 1). Relapse rates in the years beyond a successful quit attempt and continued abstinence at 12 months were included[15]. The model consists of five health states: no lung cancer (quit smoking), no lung cancer (stay smoking), early lung cancer (stage I or II), advanced lung cancer (stage III or IV), and death. Individuals will either continue or quit smoking at 12 months following either intervention and be allocated to 'no lung cancer' in the first annual cycle. Next they are dispersed into the various pathways or health states according to certain probabilities (Table 1). 'Tunnel' features have been built into the model for lung cancer states to ensure that the risk of cancer progression or death is dependent upon the duration since diagnosis. Tunnel states are a 'time in state' feature that provides a memory function to Markov models. Health state rewards and transition probabilities can be altered for each cycle patients spend in the tunnel state [16]. The model is calculated by summing the expected (mean) values at each tree node for each course of action and aggregates the longer-term health outcomes and costs for the two intervention strategies.

**Table 1:** Data parameters used in model: description, base case estimate, range tested in one-way sensitivity analyses and sources

Parameter description	Base estimate	Range tested	Sources
Quit rates: 12-month continuous abstinence			
a) Genetic Test	11%	7-22%	[12]
b) Usual treatment	6%	3-12%	[17]

Relapse rate after 12-month quit	10% in years 2-6, 4% after^1^		[15]

Lung cancer incidence	Annual from age 40, e.g., 0.0018024 at age 65 years^1^		[32]

Relative risk of lung cancer in heavy smokers compared to general population	6.609 and		[18]

Relative risk of lung cancer in ex-smokers compared to general population	Annual from 5-year age group by time since quit e.g, ages 50-55 years RR = 4.75^1^		

Survival/mortality rates (background population)	Annual by age e.g, age 65 annual dying rate = 0.00936^1^		ABS Life Tables 2005-07^2^

Survival rates of lung cancer	Annual survival at 1 year 36% to 12% at 5 years		AIHW [33]

Proportion of			
a) early lung cancer	20%	13-23%	[33], authors assumption^3^
b) adv lung cancer	80%	77-87%	

Utility scores			
a) Early stage lung cancer (I&II)	0.73	0.69-0.83	[23,34]
b) Adv stage lung cancer (III&IV)	0.66	0.30-0.76	[23,34]
c) No lung cancer	1	-	authors assumption

Lung cancer healthcare costs			
a) Early lung cancer 1st year (NSCLC only)	44,274		[35,36]
b) Adv lung cancer + SCLC 1^st ^year	27,057	All ± 30%	[35,36]
c) Ongoing costs (stable disease)	7,115		[36,37]
d) Progressive disease	10,945		[36,37]
e) Terminal care (final year)	9,961		[36,37]

1. Tables are used rather than one point estimate to account for different values that change over time. Values will alter when individuals age.

2. Epidemiological data and cost data are from slightly different years; data from these life-tables are from 2005-2007 while costs in 2009 AU$.

3. A proportion of approx. 8% of lung cancers are 'unstaged' but to avoid losing these people in the model, the proportion unstaged was assumed to be equally split into early and advanced disease groups.

Abbreviations: ABS - Australian Bureau of Statistics, AIHW - Australian Institute of Health and Welfare, NSCLC - non-small cell lung cancer, SCLC - small cell lung cancer.

### Description of the two strategies

We compared a usual smoking-cessation program with an alternative involving the usual smoking-cessation program and a genetic test some point after (e.g., 6 weeks) completing the program (as per McBride *et al. *2002 [12]). The benefit of this test is to decrease the likelihood that an individual will relapse and begin smoking again as measured by relapse rates at 12 months.

In our model, we assumed our cohort were 50 year old heavy-smoking men and women (> 20 cigarettes per day) who presented to their GP, and were willing to participate in a smoking-cessation program. The usual smoking-cessation program comprised of GP advice, telephone counselling and nicotine replacement therapy (NRT) administered over 12 weeks (Table 2). Although there are new pharmacological therapies available that show superior smoking-cessation rates (i.e., bupropion, varenicline 12-19% [6]) than those for NRT (6% [17]), NRT is widely available, accepted in most countries and has only minor adverse side-effects or contraindications. Furthermore, it is cost-effective and recommended first-line therapy in clinical practice guidelines for smoking cessation in Australia[6]. The genetic testing option is assumed to include a blood sample and assessment of other lung cancer risk factors. A second doctors' visit is required so that the doctor can communicate the test results and overall risk assessment to the individual who is also presented with a booklet explaining the test results.

**Table 2:** Intervention components and unit costs for usual smoking-cessation (USC) and USC plus genetic test

			Qty	Unit cost	2009 AU$	Source
USC (NRT with telephone counselling)						
1	GP visit	Standard 5-25 minutes	1	21.00	21.00	[6] MBS item 53
2	Patches	1st step - 21 mg/6 pkts	6	47.95	287.70	Retail pharmacy^1^
	(10 weeks)	2nd step - 14 mg/2 pkts	2	27.95	55.90	
		3rd step - 7 mg/2 pkts	2	27.95	55.90	
3	Phone counselling	Initial + 4 sessions	5	75.74	378.70	DVA, $119.75 initial then $83.70/hr
4	Booklet	Self-help materials	1	2.90	2.90	[6]
				Total	802.10	
USC + Genetic test						
1	USC as above				802.10	
2	Clinic visit	Standard 5-25 minutes	2	21.00	42.00	MBS online schedule, item 53
3	Test	Blood sample, transfer to lab and analysis	1	311.00	311.00	[13]
4	Test booklet	Explains results of gene test	1	2.90	2.90	Assumption - same for quit booklet
				Total	1158.00	

1. Price is based on the sale price at a large, urban pharmacy in Brisbane, AUD in 2008. Prices will vary according to conditions and place of purchase (e.g., online pharmacy suppliers vs. neighbourhood pharmacies). Note that the choice of the appropriate price does not impact on the results from the cost effectiveness analysis as the cost is common to both arms of the model.

2. Abbreviations: USC - usual smoking cessation, NRT - nicotine replacement therapy, MBS - Medicare Benefits Schedule, DVA - Department of Veteran's Affairs, pkts - packets

### Data parameters in the model

The data used to populate the model was based on published literature, national reports and government cancer statistics, however a number of assumptions were also necessary (Additional file 1, Table S1). The key parameters in the model were quit rates in the two arms and, for the genetic test arm, we have assumed that these behaviour changes have occurred regardless of the underlying properties of the genetic test. Systematic reviews and results of meta-analyses were used to inform estimates on 12-month quit rates of NRT [17] and relapse rates beyond 12 months[15]. Although it is possible to that 'natural' quitters, those needing no assistance to quit smoking, may exist in both groups, we have assumed the natural quit rate is equivalent in both arms. Risk estimates of lung cancer are dependent on gender, time since quitting and smoking frequency and were derived from a cohort study of over 463,000 US men and women[18]. Current epidemiological evidence provided information on background incidence of lung cancer by stage, mortality and survival rates of lung cancer, and all-cause mortality among smokers. To reflect changing estimates as the cohort ages, we accounted for age-dependent variables using tabulated data in our model. Table 1 lists all data estimates and tabled data in the model with their respective sources and ranges tested in the sensitivity analyses.

### Outcome measures

The measures of benefit in the evaluation were the number of quitters and quality-adjusted life-years gained (QALYs) over 35 years. The number of quitters at 12 months is also presented to highlight the shorter-term impact. The level of effectiveness of smoking-cessation enhanced with a genetic test was based on a randomised clinical trial involving 557 participants[12]. The proportion of individuals achieving continued abstinence at 12 months was 11% compared with 5% in the NRT only arm (p = 0.08). This study was chosen as it included the comparison groups most relevant for an Australian setting, that is, NRT plus counselling with or without a genetic test. McBride's study was also randomized, prospective, used an intention-to-treat analytical approach and included largely lower socio-economic smokers. Three other studies assessing the impact of genetic susceptibility on smoking-cessation [19-21] did not investigate relevant comparators including one with no control group, were non-randomized or had earlier-time quit rates. These quit rates ranged from 6-19%. Evidence for the effectiveness of NRT alone was based on a published systematic review of 136 randomized controlled trials, over 40,000 participants and yielding a summary estimate of 6%[17]. In the absence of outcomes of genetic testing on smoking-cessation beyond 12-months, we assumed relapse rates from the literature were equivalent in the two arms.

The QALY is a generic outcome measure preferred for use in economic evaluations combining survival time adjusted for quality of life. A structured literature review was undertaken to locate recent preference-based quality of life scores (or utility weights) for lung cancer. Eleven studies from 1997-2008 were uncovered. The utility weights used in the present study were based on direct utility assessment using standard gamble interviews [22] and a second study that used the EuroQol 5D questionnaire [23]. These studies were chosen because utilities were available for advanced/early stage and stable/progressive lung cancer, were more likely to reflect current treatment patterns and side-effects [22] and reported a range of scores to acknowledge uncertainty[22,24].

### Analysis

The costs and outcomes for the two options were combined into incremental cost-effectiveness ratios (ICERs), that is, incremental cost per quitter and incremental cost per QALY gained. The ratios are calculated as follows:

$$ICER=\frac{C_{GT}-C_{USC}}{E_{GT}-E_{USC}}$$

Where C = costs, E = effects (QALYs or quitters), GT = genetic testing arm and USC = usual smoking-cessation arm and represent the additional costs per health benefit of the genetic testing component. Our analysis took a payer perspective when measuring and valuing resources used for the two options. This included two payers; the consumers and health providers and the analysis aggregated the costs from both payers. Direct costs borne by the consumers (smokers) included over-the-counter NRT and the genetic test (Table 2). Health providers primarily bear the cost of lung cancer diagnosis, treatment and follow-up care and health care counselling and advice during smoking-cessation programs. Costs and effects were discounted at 5% and brought forward to 2009 Australian dollars using the health component of the Consumer Price Index.

### Sensitivity and scenario analyses

Threshold analyses were undertaken to separately determine at what quit and relapse rates the genetic testing arm was cost-effective. To determine if any variables were primarily driving the cost-effectiveness results, one-way sensitivity analyses on all parameters were undertaken (Table 1). Of particular importance is the 12 month quit rate of 11% following a genetic test compared with the 6% base quit rate [17]. The stability of the results to the quit rates was explored by examining quit rates of 7%, 15% and 22% for the genetic test option, and 3%, 9% and 11% for the usual smoking-cessation program. Relapse rates were also halved to explore the optimistic scenario commonly used in previous work [4,25,26]. Break-even analysis was used to identify the quit rate required for the genetic test to be cost-effective compared with usual smoking-cessation.

A probabilistic sensitivity analysis was also performed, re-sampling from nominated distributions of data inputs through 10,000 iterations. Beta distributions were assigned to probabilities (e.g., quit and relapse rates, health state transitions) and gamma distributions were assigned to cost variables because these are often right-skewed. The simulated mean ICER (QALYs) with 95% confidence intervals (CI) was generated. Finally, to assess the structural uncertainty of our model, we re-examined the model for men and women separately because it is well known that men are heavier smokers and have higher risks of lung cancer compared to women. We also explored the model for all persons starting at age 30 and 60 years. During our analyses, we assumed a willingness-to-pay ICER threshold of $20,000 per QALY gained to guide the interpretation of the findings, a level in keeping with higher-end cost-effectiveness ratios found in previous evaluations of smoking-cessation programs[5].

## Results

The cost-effectiveness results suggest that for smokers offered a smoking cessation program with a genetic test, an additional $300 on average is incurred compared with a usual smoking-cessation program (Table 3). For the smoking-cessation program with the genetic test, the corresponding mean discounted QALYs were 14.288 compared with 14.298 QALYs for usual smoking cessation. Compared with usual smoking-cessation, the genetic testing strategy produced an incremental cost-effectiveness ratio of AU$27,572 per QALY gained (Table 3) over 35 years.

**Table 3:** Results of incremental cost-effectiveness ratios (ICER) in base case and probability sensitivity analysis

Short-term (at end of 12-months)	NRT + counselling	NRT + counselling + genetic test	Difference	
Cost for 1000 persons in each arm	$802,100	$1,158,000	$355,600	
Number of quitters @ 12 months	60	110	50	
ICER - per quitter @ 12 months	-	-	$7,112	

Long-term (at end of 35 years)				

Mean cost per person	$6,600	$6,900	$300	
QALYs gained per person	14.288	14.298	0.0109	
ICER - QALYs gained per person	-	-	$27,572^1^	

Monte Carlo simulated ICERs	Incremental costs^2^	Incremental QALYs	ICERs (QALYs)	(95% CIs)

Base case ICER	$299.46	0.0109	$34,687^3^	($12,483, $87,734)
Initial cohort aged 30 years	$341.69	0.0032	$133,409	($53,502, $361,376)
Initial cohort aged 60 years	$275.66	0.0126	$27,601	($8,783, $73,948)
Men only (aged 50 years)	$286.23	0.0130	$27,182	($9,200, $70,783)
Women only (aged 50 years)	$334.53	0.0049	$46,408	($17,199, $118,383)

1. ICER of simple average results - single mean cost and effect differences.

2. Statistically significantly different mean costs and effects between groups (p < 0.001)

3. Average ICER of 1,000 simulations, not ICER of average results.

These results suggest an ICER above the threshold level of AU$20,000 per QALY gained. We found that the 12-month quit rate would need to be at least 12.4%, or that the long-term relapse rate needed to be 12% lower, for the genetic testing strategy to be as cost-effective as the usual smoking-cessation strategy (Additional file 1, Figures S1 & S2). The predicted proportions of the cohort who quit or relapsed for both strategies by age are highlighted in Additional file 1, Figure S3 and similarly for those with early and advanced lung cancer in Additional file 1, Figure S4.

Over a short-term 12-month period, for every 1000 individuals undertaking smoking-cessation enhanced with a genetic test, an additional cost of $355,600 would result in 50 additional quitters or $7,112 per additional quitter over 12 months compared with usual smoking-cessation (Table 3).

### Sensitivity & scenario analyses

**Figure 2:**
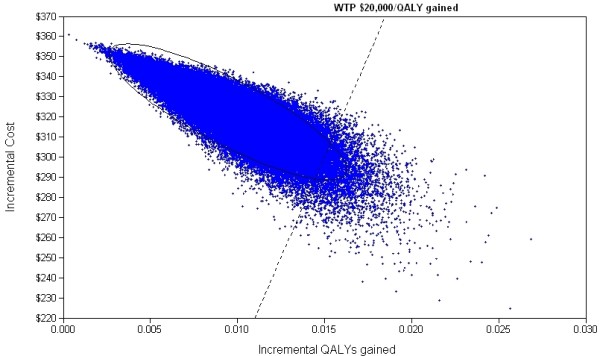
**Scatterplot of incremental cost per QALY gained with 95% ellipse and willingness-to-pay (WTP) AU$20,000 per QALY gained**.

One-way sensitivity analyses indicated that the model was highly volatile to changes in quit rates in both intervention arms and the relative risks of lung cancer for smokers and ex-smokers (Additional file 1, Figure 5). Under more favourable scenarios, when the quit rate of 22% for genetic testing was used, the ICER was $2,203 per QALY or when the cost of a genetic test was halved, as may be the case if the technology became less expensive over time, the ICER was $8,247 per QALY. In a two-way analysis, when the quit rates were 22% and 12% for the genetic testing and usual care arms respectively, the ICER was $5,553 per QALY. Probabilistic sensitivity analyses indicated a mean ICER of $34,687 per QALY gained (95% CI $12,483, $87,734) (Table 3). Our simulated base ICER of $34,687 per QALY gained was somewhat higher than our simple 'expected value' base ICER of $27,572 because the simulated ICER is calculated from the average of 10,000 mean costs and mean effects based on several uncertain parameters with their assigned distributions while the simple ICER is based on fixed mean cost and effect estimates. The simulated ICER sampling mean estimates are the correct and preferred 'expected values' for the model. At a willingness to pay of $20,000 per QALY gained and using conservative estimates, the probability that the genetic test option is a cost-effective addition to the usual intervention is 20.6% (Figure 2) compared with 99.9% using more optimistic quit rates for the two arms.

The cost-effectiveness ratios were lower than our base case when applied to men only $27,182 per QALY (95%CI $9,200, $70,783) and higher for women $46,408 per QALY (95%CI $17,199, $118,383) (Table 3). When we assessed the model with younger initial cohort of 30 year olds, the cost per QALY ratios increased to $133,409 (95%CI $53,502 $361,376) and for 60 year olds, decreased to $27,601 (95%CI $8,783, $73,948) (Table 3). If it was assumed that the relapse rate is halved in both strategies (i.e., 5% relapse from years 2-6, 2% thereafter), the mean ICER per QALY gained was $18,623 (95%CI $5,897, $49,228). The relapse rate would need to be zero in both arms, and the quit rate for genetic-testing option at least 18%, for the genetic-testing option to have lower costs and higher effects than usual smoking-cessation. Alternatively, keeping the relapse rate at our base level (10% years 2-6, 4% thereafter), the quit rate for the genetic-testing option needs to be at least 29% to dominate the usual smoking-cessation option.

## Discussion

The purpose of this paper was to examine the potential cost-effectiveness of smoking-cessation via NRT enhanced with genetic information on lung cancer risk using a dynamic model and up-to-date data estimates. Our results suggest that using the 12 month quit rate reported in a previous trial, [12] the genetic testing option is unlikely to be cost-effective at a threshold of $20,000 per QALY gained. The genetic test option would need to achieve a 12-month continuous quit rate of 12.4% or more for it to be a cost-effective addition to NRT and counselling treatment alone. Alternatively, the genetic testing option would need to achieve relapse rates 12% lower than those for usual smoking-cessation. Although our base ICER $34,687 per QALY is higher than the $20,000 threshold, we emphasize that the high volatility in the model estimates means that the genetic test option could easily become cost-effective if further evidence supported mildly more optimistic quit or relapse rates. However, overall we found very small differences in cost between the two options over a period of 35 years and similarly for differences in effects. The model was very sensitive to small changes in critical variables such as the 12-month quit rates and relapse rates after 12-months, hence the results are unstable. Further research on smoking-cessation quit rates following genetic testing is needed to improve the validity of the values used in our model and reduce the uncertainty of our findings.

Our ICER of $34,687 per QALY gained would be considered cost-effective in relation to accepted thresholds for pharmacological health care treatments in Australia [27]. However, given that we are not assessing a pharmaceutical and that other ICERs of smoking cessation options are among the lowest of all health interventions, we used a $20,000 acceptable threshold [5]. Several studies have shown better health outcomes and cost-savings are possible for varenicline,[26,28] bupropion [28] and community pharmacy-led [29] programs. In this context, it would seem that our genetic testing strategy is a relatively poor investment. However, with at least 30 published studies providing evidence that a wide variety of smoking-cessation interventions are cost effective, these findings may be less favourable because most studies have overestimated long-term effectiveness due to assumptions made with smoking relapse rates or evaluation time frames being too short[30]. In our study, the use of improved epidemiological data on the risk of developing lung cancer separating risk estimates by gender, time since quitting and heavy/light smoking patterns [18] should provide more precise cost-effectiveness estimates[31].

Traditionally, men are heavier smokers than women and their relative risk of lung cancer is higher. This explains the lower (more favourable) ICERs for men because they have relatively higher numbers of life-years to gain from stopping smoking[5,25]. However, due to the large uncertainty in the model, differences between men and women were tenuous. The benefits of smoking-cessation can occur at any age of quitting however, the risk of lung cancer among ex-smokers versus non-smokers remains elevated even after more than 40 years of cessation[9]. Our findings are in contrast to other studies where smoking-cessation among younger cohorts has more favourable cost-effectiveness than for older cohorts. Our opposite finding results from the fact that a given percentage of people who quit at 12 months are assumed to relapse each year, meaning that some (younger) people will start smoking again before the benefits of not smoking (avoided cancer) are realized. Additional research is need to identify whether relapse rates for younger smokers would in fact remain low after receiving positive results from a genetic test.

When our model was re-assessed for 30 year olds, the long-term effects were severely eroded due to discounting and relapse rates. Therefore, the overall effectiveness was very small, inflating the cost-effectiveness ratio. This finding would indicate that the genetic testing arm is potentially suitable only in older (at least 50 year olds), long-term smokers or that the NRT and counselling needs to be repeatedly offered in relapsed smokers [30] and is not cost-effective as a one-off intervention.

Our choice of relapse rates is an important variable in our model both in terms of the values used, which were taken from a meta-analysis,[15] and the 35 year model duration. These have a combined effect of having a cumulative lifetime relapse of 78% (subject to some quitters dying before they are able to relapse), considerably higher than studies using Markov models with lifetime relapse rates of 35%[4,25]. When the base case relapse rates were halved and closer to those used previously, the cost-effectiveness ratios were substantially lower; $18,623 (95%CI $5,897, $49,228).

While our model was responsive to an ageing cohort and other time-dependent variables, some limitations are apparent and a number of assumptions were necessary. Data estimates are based on those available in published randomized controlled trials and may not reflect real-world practice (e.g., overestimated effects or compliance from experimental trial data). It is acknowledged that many individuals permanently cease smoking on their own accord with no psychological or pharmacological assistance. The present study examines the relative effectiveness of a smoking cessation program compared with a smoking cessation program given in conjunction with a genetic test. Extensive sensitivity analyses explored parameter uncertainty and aspects of the structural uncertainty (e.g., different cohort profiles). We relied on a single, randomized clinical trial by McBride *et al*. (2002) for a critical estimate, quit rate at 12-months following the genetic test[12]. This study was US-based and involved a largely African-American lower-socioeconomic cohort. Arguably, McBride *et al.'s *sample of mostly lower-socioeconomic smokers may be a difficult group to intervene in but likely to be relevant and generalisable to other settings like Australia where a higher proportion of disadvantaged people also smoke. Potentially adverse consequences of genetic testing include emotional distress, concerns about discrimination and implications for telling family members positive results. These issues were omitted from our analysis. Our results relate to QALYs gained from preventing lung cancer onset and we did not incorporate improved survival gains due to the potential avoidance of other major diseases linked to smoking (e.g., heart disease, COPD, diabetes). Again, the impact is that our effects may be underestimated and overall ICERs conservative. A further limitation of the study was the omission of the potential implications of interactions between the level of susceptibility, test properties and quit rates that may impact on the cost-effectiveness findings, introducing further uncertainty. Based on McBride's findings, 33% of the participants in the GT arm had a positive genetic test for the missing gene GSTM1 for elevated susceptibility to lung cancer. However, quit rates in these participants were similar to those with negative tests and therefore behavior change was not hindered by the GT results. This finding is supported by our own pilot work with further results on this issue forthcoming.

Lung cancer is the leading cause of cancer death in many developed countries and the prognosis is poor with a 1-year survival of 34% and 5-year survival of 12%[32]. Although the risk of lung cancer is small in individuals with 'at risk' genotypes, lung cancer is a common cancer and therefore those with a genetic susceptibility affects a high absolute number of smokers[8]. Further research on genetic susceptibility and molecular epidemiology in lung cancer alongside overall risk assessments [7] remains important work before public health approaches of screening, targeted smoking-cessation programs or other preventive measures are adopted[8]. At the same time, commercial availability and consumer interest in genetic testing is increasing and may create added pressure for insurance companies or governments to subsidize their costs[11]. To date, the evidence to support effective smoking-cessation by informing individuals of their own genetic risk of lung cancer is promising but weak[10,12]. Genetic testing strategies rely on successful doctor-patient communication and must be ethical, results accurately conveyed and understood by patients[11].

## Conclusion

In certain circumstances, specifically, if a smoking-cessation program delivering a genetic test, NRT and counselling produced a 12-month quit rate of at least 12.4% then it would represent a potentially sound health care investment for 50 year old heavy smokers. Overall, our findings showed that a genetic test option in addition to the use of NRT and counselling would produce very similar costs and effects than NRT and counselling alone. Further research on the quit rates at 12 months and beyond following a genetic testing strategy is required to strengthen our findings.

## List of abbreviations

CI: Confidence interval; COPD: Chronic obstructive pulmonary disease; GT: Genetic test; ICER: Incremental cost-effectiveness ratio; NRT: Nicotine replacement therapy; QALY: Quality adjusted life years; SNP: Single nucleotide polymorphisms; USC: Usual smoking cessation

## Competing interests

Dr Robert Young is a Scientific Advisor to Synergenz BioSciences who sponsored separate, but related, projects in lung cancer genetics and risk assessment scores.

Dr L Gordon, N Hirst and Dr P Brown declare that they have no competing interests.

## Authors' contributions

LGG: performed the systematic review, data analyses, interpretation and drafted the manuscript.

NGH: assisted with systematic review, data analyses, interpretation and presentation of the findings and manuscript writing.

RPY: provided clinical expertise, idea conception and intellectual input and interpretation of the overall findings

PMB: provided senior public health expertise, intellectual input and guidance during manuscript writing.

All authors have contributed substantively to writing the manuscript and have approved the final version.

## Supplementary Material

Additional file 1 **Figure S1**: Threshold analysis of quit rate required for the genetic test strategy to have equivalent net benefits as usual smoking-cessation, at a willingness to pay (WTP) of $20,000. **Figure S2**: Threshold analysis of proportion of relapse rate required for the genetic test strategy to have equivalent net benefits as usual smoking-cessation, at a WTP of $20,000 **Figure S3**: Proportion of cohort who are quitters or relapsers, by age and genetic test or usual smoking-cessation arms. **Figure S4**: Proportion of cohort who develop early or advanced lung cancers, by age and genetic test or usual smoking-cessation arms. **Figure S5**: Results of one-way sensitivity analyses on key parameter values showing change in base case incremental cost per QALY ratio **Table S1 **- Model assumptions Click here for file
