# Supplementary material for: Within a smoking-cessation program, what impact does genetic information on lung cancer need to have to demonstrate cost-effectiveness?
Source: Cost Eff Resour Alloc. 2010 Sep 16;8:18. doi: 10.1186/1478-7547-8-18 (PMC2949618; doi:10.1186/1478-7547-8-18)
Supplement: Additional file 1 — Figure S1: Threshold analysis of quit rate required for the genetic test strategy to have equivalent net benefits as usual smoking-cessation, at a willingness to pay (WTP) of $20,000. Figure S2: Threshold analysis of proportion of relapse rate required for the genetic test strategy to have equivalent net benefits as usual smoking-cessation, at a WTP of $20,000 Figure S3: Proportion of cohort who are quitters or relapsers, by age and genetic test or usual smoking-cessation arms. Figure S4: Proportion of cohort who develop early or advanced lung cancers, by age and genetic test or usual smoking-cessation arms. Figure S5: Results of one-way sensitivity analyses on key parameter values showing change in base case incremental cost per QALY ratio Table S1 - Model assumptions [file 1478-7547-8-18-S1.DOC]

**Additional File** **1**

**Figure S1 - Threshold analysis of quit rate required for the genetic test strategy to have equivalent net benefits as usual smoking-cessation, at a willingness to pay (WTP) of $20,000.**

Interpretation: The genetic test strategy needs to have a quit rate of at least 12.4% at 12 months to produce superiority to the usual smoking-cessation option.

**Figure S2 - Threshold analysis of proportion of relapse rate required for the genetic test strategy to have equivalent net benefits as usual smoking-cessation, at a WTP of $20,000**

Interpretation: The genetic test strategy needs to have a relapse rate up to 88% than that for usual smoking-cessation to be a superior option.

**Figure S3 - Proportion of cohort who are quitters or relapsers, by age and genetic test or usual smoking-cessation arms.**


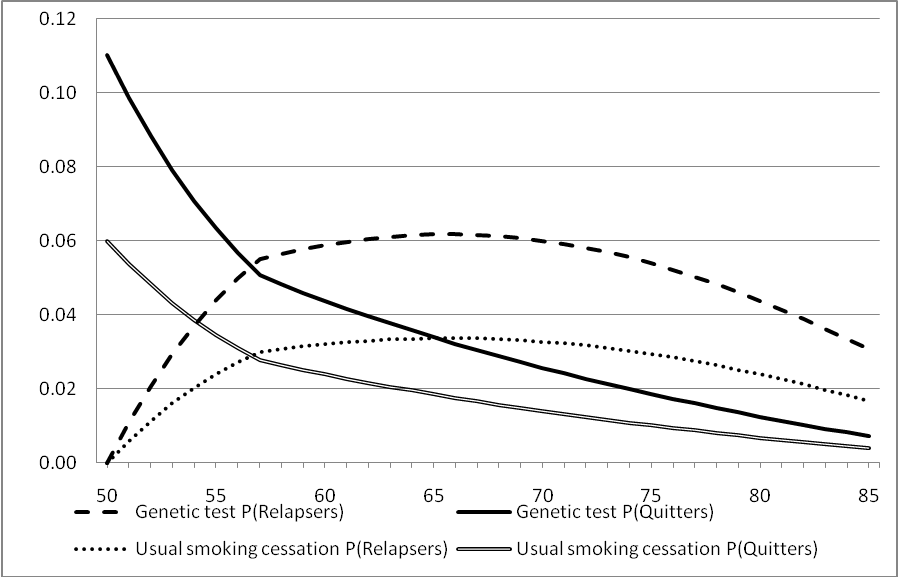


**Figure S4 - Proportion of cohort who develop early or advanced lung cancers, by age and genetic test or usual smoking-cessation arms.**


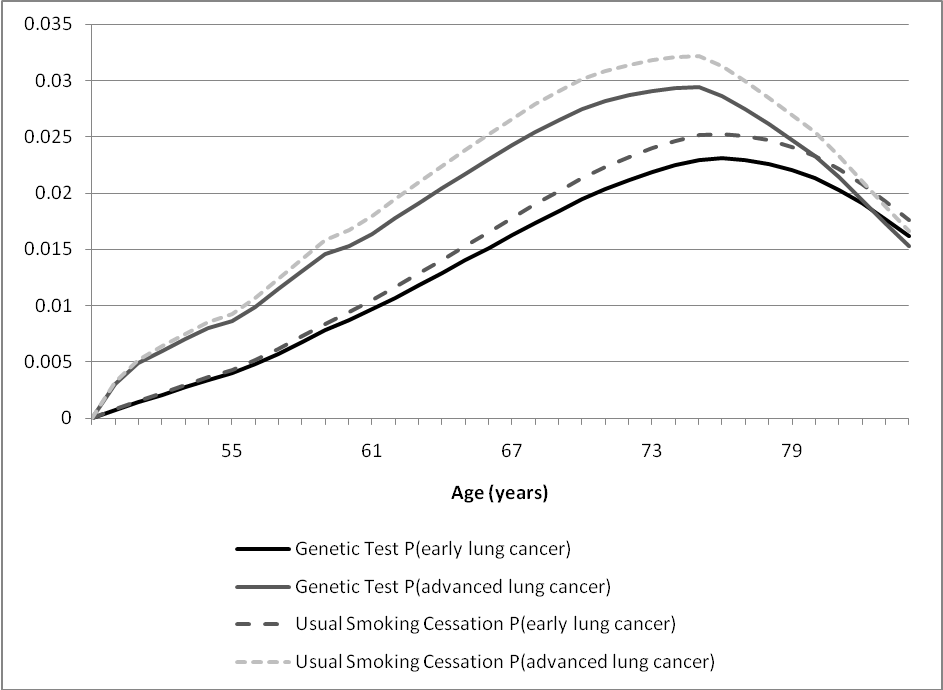


**Figure S5 - Results of one-way sensitivity analyses on key parameter values showing change in base case incremental cost per QALY ratio**


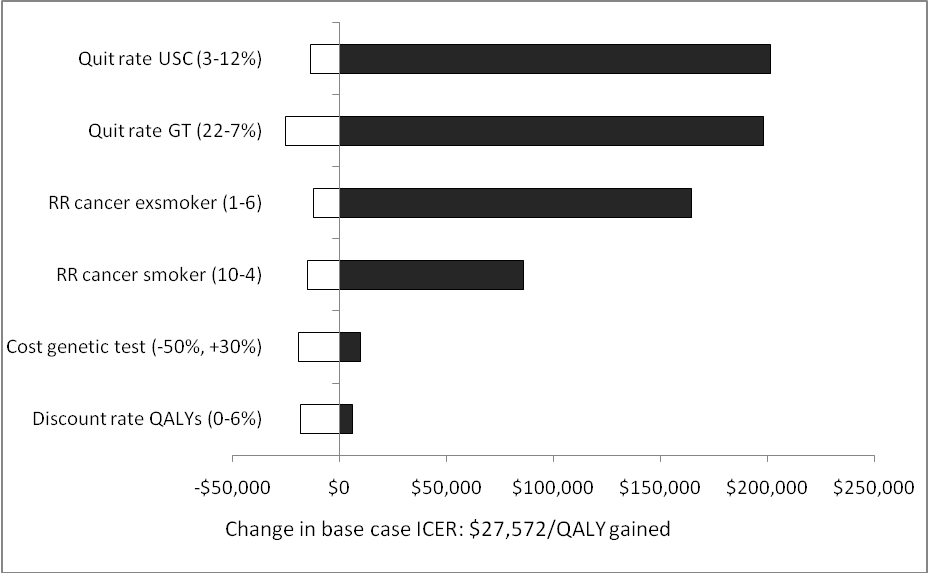


Abbreviations: GT – genetic testing option, USC – usual smoking cessation option, RR – relative risk, QALY – quality-adjusted life-year

Interpretation: The base case ICER of $25,572 per QALY is highly sensitive to the quit rates of both options and the relative risk estimates of lung cancer for smokers and exsmoker. More precise estimates on these parameters are needed to confirm the ICER findings here.

**Table S1 - Model assumptions**

|  | In model structure |
| --- | --- |
| 1 | The structure of the genetic test arm is modelled on a program described in a randomized control trial by McBride *et al.* (2002) [1] |
| 2 | Background effects from Australian National Tobacco Campaigns are equal in each arm and not assessed |
| 3 | Population characteristics - initial cohort are heavy smokers only (>20 cigarettes per day) therefore are more addicted, require more help and may have had previous quit attempts |
| 4 | Genetic test facilitated within GP setting with smoker having started NRT + counselling - 2 visits required (1 for test & 1 for communicating the test results) |
| 5 | NRT and proactive counselling protocols/resources are recommended by Australian smoking cessation guidelines [2] |
| 6 | 100% compliance with NRT and genetic testing with both intervention arms |
| 7 | NRT duration is 10 weeks as per recommendations - single course only |
| 8 | Behaviour change occurs irrespective of genetic test performance. For information, based on the authors (R Young) work, in the average smoking population about 50% of smokers are “moderate risk”, 30% are high risk and 20% are “very high” risk, where moderate equates to the average smoker. No one is at low risk. The sensitivity and specificity varies according to the score (range 1-12). [3] |
|  | In model parameters |
| 1 | Quit rates for NRT were pooled as there are no significant differences between the methods available (gum, patch, inhaler, tablets) [4] |
| 2 | Relapse rates in the base case were identical for the two strategies |
| 3 | Price of gene test are comparable in Australia (UK based in model £150) [5] |
| 4 | Prices of NRT option are for patches, these are most widely used form of NRT |
| 5 | Full health is attributed as pay-offs to smokers and ex-smokers who do not have lung cancer (Utility=1.0) |
| 6 | Number of quitters at the end of the model time frame may have died from causes other than lung cancer |
| 7 | Proportion of individuals with unstaged lung cancer equally divided into early and advanced cancer |

**References for Table S1**

[1] McBride CM, Bepler G, Lipkus IM*, et al.* Incorporating genetic susceptibility feedback into a smoking cessation program for African-American smokers with low income. *Cancer Epidemiol Biomarkers Prev* 2002;**11**(6):521-528.

[2] Royal Australian College of Physicians. Smoking Cessation Guidelines for Australian General Practice. 2007.

[3] Young RP, Hopkins RJ, Hay BA*, et al.* Lung cancer susceptibility model based on age, family history and genetic variants. *PLoS ONE* 2009;**4**(4):e5302.

[4] Stead LF, Perera R, Bullen C*, et al.* Nicotine replacement therapy for smoking cessation. *Cochrane Database Syst Rev* 2008;**23**(1):CD000146.

[5] Heitjan DF, Asch DA, Ray R*, et al.* Cost-effectiveness of pharmacogenetic testing to tailor smoking-cessation treatment. *Pharmacogenomics J* 2008;**8**(6):391-399.
